# Myoelectric Pattern Recognition Using Gramian Angular Field and Convolutional Neural Networks for Muscle–Computer Interface

**DOI:** 10.3390/s23052715

**Published:** 2023-03-01

**Authors:** Junjun Fan, Jiajun Wen, Zhihui Lai

**Affiliations:** 1 College of Computer Science & Software Engineering, Shenzhen University, Shenzhen 518060, China; 2 Guangdong Key Laboratory of Intelligent Information Processing, Shenzhen University, Shenzhen 518060, China; 3 Guangdong Laboratory of Artificial-Intelligence and Cyber-Economics (SZ), Shenzhen University, Shenzhen 518060, China

**Keywords:** muscle–computer interface, surface electromyography, myoelectric pattern recognition, Gramian angular field, convolutional neural networks

## Abstract

In the field of the muscle–computer interface, the most challenging task is extracting patterns from complex surface electromyography (sEMG) signals to improve the performance of myoelectric pattern recognition. To address this problem, a two-stage architecture, consisting of Gramian angular field (GAF)-based 2D representation and convolutional neural network (CNN)-based classification (GAF-CNN), is proposed. To explore discriminant channel features from sEMG signals, sEMG-GAF transformation is proposed for time sequence signal representation and feature modeling, in which the instantaneous values of multichannel sEMG signals are encoded in image form. A deep CNN model is introduced to extract high-level semantic features lying in image-form-based time sequence signals concerning instantaneous values for image classification. An insight analysis explains the rationale behind the advantages of the proposed method. Extensive experiments are conducted on benchmark publicly available sEMG datasets, i.e., NinaPro and CagpMyo, whose experimental results validate that the proposed GAF-CNN method is comparable to the state-of-the-art methods, as reported by previous work incorporating CNN models.

## 1. Introduction

Electromyographic signals (EMGs), which provide representations of electrical potential fields, are crucial biomedical signals produced by membrane depolarization of the muscle fibers. The well-established surface electromyography (sEMG) is a kind of EMG signal acquired from the skin surface [1]. Traditionally, sEMG signals are used to analyze muscle activities and assess functional diagnosis in clinical settings [2]. Recent studies have pointed out that sEMG can be exploited as a feasible input modality for a computer to generate a muscle–computer interface (MCI) with a wide range of applications, such as controlling prosthetics [3], human–computer interfaces [4,5], and sign language recognition [6]. Saponas et al. achieved an accuracy of 78.0% when classifying wrist and finger flexion via forearm sEMG sensing [7]. Huang et al. used an sEMG wristband to detect hand–smartphone interaction, obtaining an accuracy of 82.9% for classifying fine-grained thumb gestures such as left swipe, right swipe, tap, and long press [5]. The above studies have demonstrated the feasibility of using forearm electromyography for MCI. As the performance of traditional vision-based human–computer interaction (HCI) methods depend on the constrained environment, e.g., indoor environment, stable lighting conditions, and simple backgrounds [8], myoelectric pattern recognition is a ubiquitous HCI method that has strong robustness to the environment. 

The identification of sEMG signals with regard to various motion patterns is the most important task for MCI. Meanwhile, conventional machine learning techniques have shown promising performance in a variety of applications [9]. However, difficulties in extracting the meaningful handcrafted and domain-dependent features still exist in the application of sEMG signal analysis. Recent studies in deep learning (DL) [10] show that deep networks are able to intrinsically extract important features and better performances on large-scale datasets [11,12,13] can be achieved. With advances in DL, many studies have explored the possibility of myoelectric pattern recognition without handcraft features, which overcomes the drawbacks of conventional machine learning methods by allowing the transformation of data to a more abstract representation [14,15,16]. The main popular DL architectures that are applied to myoelectric pattern recognition include deep neural networks (DNNs), deep recurrent networks (DRNNs), convolutional neural networks (CNNs), autoencoders (AEs), and deep belief networks (DBNs) [17]. Park et al. [15] built a CNN-based classification model that obtained promising results for six common gestures, which were 12–18% higher than the conventional machine learning techniques. Most of the approaches are data-driven and employ the sEMG data directly as input [17]. However, sEMG is a kind of nonstationary micro-electrical signal, which makes it hard to extract the patterns when directly using the raw sEMG signals as the model input in deep learning. Therefore, it is especially important to adjust the input of the deep model appropriately according to the characteristics of electromyographic signals. 

To address the problem caused by using sEMG data as the direct input for CNN learning [18,19], we propose conducting sEMG signal feature extraction after signal morphology conversion under a DL framework. 

Gramian angular field (GAF) encodes one-dimensional data into a two-dimensional symmetrical matrix with the matrix elements defined in polar coordinates [20]. Existing works exploited the potential of using GAF encoding time-series data into 2D images for CNN classification in many applications, such as multivariate time-series image forecasting [21], fiber Gragg grating sensing [22], and electromagnetic modulation signal classification [23]. Inspired by these works, we found that the GAF method can reflect the combination of signals at different positions of one-dimensional data in the form of two-dimensional data. Therefore, it would be feasible to use the GAF method to encode multichannel sEMG signals as images, which can make the feature space more expressive by representing relationships between the values in different sensor channels rather than treating each channel independently. Based on the GAF method and a CNN-based model, we designed a two-stage architecture, GAF-CNN, consisting of a 2D representation stage and a CNN-based classification stage, to improve the accuracy of myoelectric pattern recognition. GAF-CNN employs the GAF method to encode the instantaneous values of multichannel sEMG signals to generate sEMG-GAF images. A deep convolutional neural network (DCNN) model is proposed for automatic sEMG-GAF image feature extraction and classification. It is well known that DCNN can preserve the neighborhood connection and spatial characteristics among local regions [24]. Considering the small scale of the dataset, a DCNN framework is constructed using an input layer, five convolutional layers, and three fully connected layers. Batch normalization is employed instead of using max-pooling layers after convolution to optimize the network. A dropout strategy is adopted on the two fully connected layers to avoid overfitting. This work makes the following contributions:

1. A two-stage architecture consisting of a 2D presentation stage and a CNN-based classification stage is proposed for myoelectric pattern recognition, where the raw sEMG signal is converted to a suitable 2D representation for better CNN feature extraction.
2. A 2D presentation method employing GAF is proposed to encode instantaneous multichannel sEMG signals in image form, and the rationale of the proposed method is given.
3. Evaluations on four benchmark sEMG datasets show that GAF-CNN has outstanding classification performance compared with the state-of-the-art CNN-based myoelectric pattern recognition methods.

The remainder of this paper is organized as follows. Section 2 presents the related work. Section 3 describes the GAF-CNN method for gesture recognition and explains its rationale. Section 4 presents our experimental results. Section 5 states our conclusions.

## 2. Related Work

Recent studies show the promise of machine learning in feature extraction, which has frequently been used in myoelectric pattern recognition for human movement identification [17]. Conventional machine learning pipelines include data acquisition, feature extraction, mathematical modeling, and inference. Frequently used feature extraction methods for myoelectric pattern recognition fall into three categories: (1) time-domain (TD) features, such as mean absolute value (MAV), root mean square (RMS), and zero crossings (ZCs); (2) frequency domain (FD) features, such as power spectrum (PS) and autoregressive coefficients; and (3) time–frequency domain (TFD) features, such as discrete wavelet transform (DWT) and wavelet packet transform (WPT) [25]. These features have been well explored and proved effective in myoelectric pattern recognition [26]. For example, Fan et al. employed 12 types of commonly used EMG features to represent object interaction and investigated their classification performance [27]. New features for gesture recognition have been proposed to improve the classification performance, such as active muscle regions based on the mapping relationship between hand movements and forearm active muscle regions [11]. Conventional classification algorithms such as support vector machine (SVM), k-nearest neighbors (k-NNs), hidden Markov models (HMMs), decision trees, random forest, linear discriminant analysis, and artificial neural networks (ANNs) have been explored to improve the performance of gesture recognition [12,13]. However, these methods cannot achieve high accuracy in real-world applications due to their poor generalization ability and complex parameter adjustment process, which depends on artificially designed extractors and professional knowledge.

In the past decade, the DL technique has undergone significant development. The deep network can intrinsically extract effective features, enabling competitive performance on challenging datasets [28,29]. With the advancements of deep learning, the in-depth features of sEMG signals have been explored and are well-studied for gesture recognition [17,26]. To use the advantages of sEMG signals and deep architecture, Zhang et al. proposed a long short-term memory (LSTM) algorithm to recognize hand gestures through multimodal sEMG data and obtain competitive classification performance [16]. Considering the pros and cons of CNN and LSTM, LSTM-CNN (LCNN) models were proposed to construct autoencoders for automatic feature extraction [30]. Notably, deep convolutional neural networks (DCNNs) can automatically extract crucial features from signals and have been used for myoelectric pattern recognition without handcraft features. Atzori et al. [18] proposed a concise CNN architecture based on five blocks of convolutional and pooling layers with better classification accuracy than classical methods. Geng et al. [19] used instantaneous sEMG images for gesture classification with 89.3% accuracy on eight types of movements. 

To further explore the correlation between specific gestures and the related sEMG signals of muscular activities, Wei et al. proposed a multi-stream CNN architecture, which divides the input data into small-size images and extracts effective features by convolutional layers before further processing with fully connected layers [31]. Considering that two-dimensional (2D) CNNs with 2D kernels cannot handle a sequence of images that carries signals over time, Chen et al. presented a three-dimensional (3D) CNN with 3D kernels to capture both spatial and temporal structures from sequential sEMG images. Experiments showed that the accuracies of 3D CNN were 18.6% higher than those of 2D CNN when the time window duration was 150 ms on a high-density sEMG dataset [32]. 

Recently, novel DL frameworks have been designed for myoelectric pattern recognition. Demir et al. [33] proposed a deep-transfer-learning-based approach with sEMG signals for human action classification, obtaining 98.65% accuracy, better than conventional methods, by fine-tuning the AlexNet model. Most of the above methods focus on improving DL models, achieving significant improvements in classification accuracy. Nevertheless, they usually use raw sEMG signals as image input directly, lacking the exploration of a good representation of the raw sEMG signals [17].

## 3. GAF-CNN

**Figure 1:**
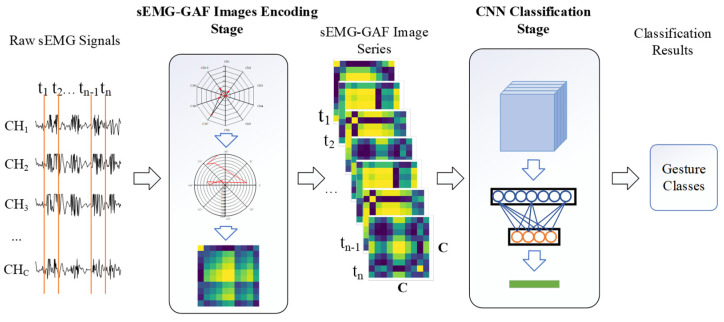
Pipeline of the proposed two-stage architecture for myoelectric pattern recognition.

We propose a GAF-CNN method that formulates the sEMG-based gesture recognition as the 2D data representation and CNN-based classification problems. The pipeline includes two stages: sEMG-GAF image encoding and CNN-based classification. In the first stage, the multi-channel sEMG values at instant sampling moments are encoded in image form based on GAF features. Hence, the value of each point in the sEMG-GAF image indicates the correlation between signals from two sEMG sensors. In the classification stage, a deep CNN model is adopted to classify the sEMG-GAF images. Figure 1 shows the pipeline of the proposed GAF-CNN method.

### 3.1. Transformation from sEMG Signals to sEMG-GAF Image

After the sEMG signals have been divided into small segments with an overlapped time window, building suitable network inputs is essential and is highly dependent on the preferred DL architecture. The CNN-based method traditionally requires an M × N gray image or an M × N × 3 RGB image as input. To this end, the sEMG signals must be reshaped as 2D or 3D matrix form. Intuitively, reshaping the sEMG signals as an image could be a solution, where a pixel of the image can be regarded as one instantaneous value from the sEMG sensor electrode [31]. This works when high-density sEMG signals are employed, since the construction of an sEMG image requires an adequate number of instantaneous values acquired by high-density sEMG sensors. On the other hand, sparse multichannel sEMG signals can be reshaped as an image form with the dimensionality of N × L, where N is the number of electrodes and L is the duration of the sliding time window [34]. Those mentioned works provide a method to generate sEMG images as input for the CNN-based method. However, the construction of an appropriate sEMG-based image that can be suitable for both high-density sEMG signals and sparse sEMG signals remains an unsolved problem. 

We present an effective approach to encode sEMG signals as images via the GAF method [20]. Unlike the use of the GAF method to convert time-series data to images, we use it for imaging instantaneous sEMG signals on sensor channels, i.e., at each sampling moment. The sEMG signals of C sensor channels are converted to an image with the size of C × C, which we call an sEMG-GAF image. In the sEMG-GAF encoding stage, one segment of sEMG signals is given by

(1) $$X=\left[\begin{array}{cccc} x_{1}^{1} & x_{2}^{1} & \ldots & x_{L}^{1}\\ x_{1}^{2} & x_{2}^{2} & \ldots & x_{L}^{2}\\ \vdots & \vdots & \ddots & \vdots \\ x_{1}^{C} & x_{2}^{C} & \ldots & x_{L}^{C} \end{array}\right],$$

where C is the number of sEMG sensor channels and L is the length of sEMG segments. 

**Figure 2:**
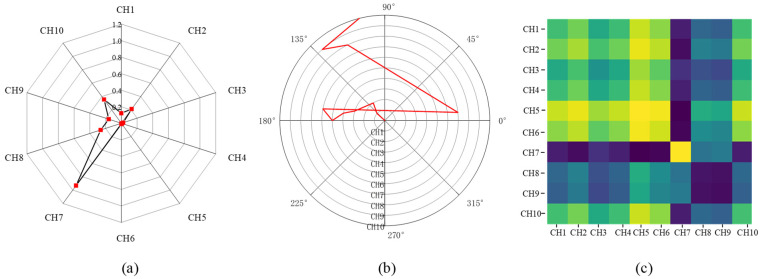
The proposed encoding procedure of GAF conversion on a selected moment: (**a**) normalized 10-channel sEMG values in radar map; (**b**) represented angular values in the polar coordinate system; and (**c**) demonstration of sEMG-GAF image after signal conversion. The different colors in (**c**) indicate relationships between the instant values in different sensors.

We rescaled X into the interval [−1, 1]:

(2) $${\tilde{x}}_{i}^{c}=\frac{(x_{i}^{c}-max\left(X\right)+\left(x_{i}^{c}-min\left(X\right)\right)}{max\left(X\right)-min\left(X\right)},$$

where c is the channel index and i is the sampling moment. Figure 2a demonstrates the radar map of the normalized 10-channel sEMG values at the selected moment.

In this way, the rescaled sEMG signals X can be represented in polar coordinates by encoding ${\tilde{x}}_{i}^{c}$ as the angular cosine:

(3) $$\left\{ \begin{array}{c} \theta_{i}^{c}=arccos\left({\tilde{x}}_{i}^{c}\right),-1\leq {\tilde{x}}_{i}^{c}\leq 1,{\tilde{x}}_{i}^{c}\in \tilde{X}\\ r=\frac{c}{C},1\leq c\leq C \end{array},\right.$$

where r is the radius that regularizes the span of the polar coordinate system. As c increases, $\theta_{i}^{c}$, which can be considered a novel representation of multichannel sEMG signals at moment i, warps among different angular points on the spanning circles in the polar coordinate. Figure 2b demonstrates the represented angular values in the polar coordinate system at a selected moment. Each angular point indicates the represented angular value of one sEMG sensor. The increment of the angular value can be used to present the relationship between two instant sEMG values. It is clear that the angular increment is bijective, as $cos\left(\phi_{i}^{c}\right)$ is monotonic when $\phi_{i}^{c}\subseteq \left[0,\pi \right]$, which means that the proposed mapping produces only one result in the polar coordinate system and has a unique inverse mapping at a given channel and moment. Additionally, unlike Cartesian coordinates, polar coordinates preserve absolute temporal relationships. Hence, polar coordinates are a good choice for the 2D representation of sEMG values.

After transforming the rescaled sEMG signal to polar coordinates, the angular increment can be calculated by the trigonometric sum between each point to identify the channel correlation of different values in sensor channels. Therefore, we define the inner product as $cos\left(\theta_{i}^{p}+\theta_{j}^{q}\right)$, where *p* and *q* are the channel indices. In this way, the sEMG-GAF image at moment i can be defined using a Gram matrix:

(4) $${\mathrm{sEMG}-\mathrm{GAF}}_{i}=\left[\begin{array}{ccc} \cos\left(\theta_{i}^{1}+\theta_{i}^{1}\right) & \ldots & \cos\left(\theta_{i}^{C}+\theta_{i}^{1}\right)\\ \cos\left(\theta_{i}^{1}+\theta_{i}^{2}\right) & \ldots & \cos\left(\theta_{i}^{C}+\theta_{i}^{2}\right)\\ \vdots & \ddots & \vdots \\ \cos\left(\theta_{i}^{1}+\theta_{i}^{C}\right) & \ldots & \cos\left(\theta_{i}^{C}+\theta_{i}^{C}\right) \end{array}\right].$$

Figure 2c demonstrates a sEMG-GAF image and different colors in the image indicate relationships between the instant values in different sensors.

To fulfill the data preprocessing requirement at the training and classification stages, the sEMG signals are formed into segments by a sliding time window. One segment of sEMG signals X with duration time L can be represented as the sequence

(5) $$\mathrm{sEMG}-\mathrm{GAFs}=\left\{{\mathrm{sEMG}-\mathrm{GAF}}_{1},\ldots ,{\mathrm{sEMG}-\mathrm{GAF}}_{i},\ldots ,GAF_{L}\right\}.$$

**Figure 3:**
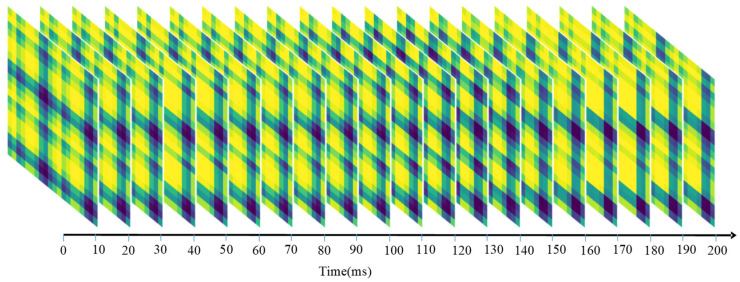
Sequential sEMG-GAF images obtained from the NinaPro DB1 dataset; 10-channel sEMG signals were sampled at 100 frames/s.

**Figure 4:**
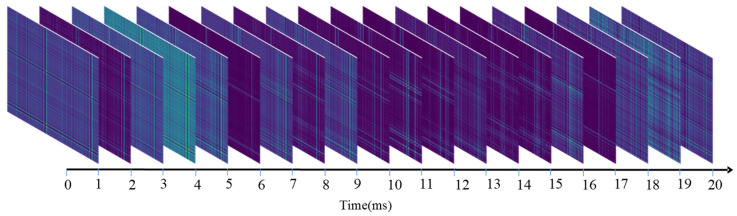
Sequential sEMG-GAF images obtained from the CagpMyo DB-a dataset; 144-channel sEMG signals were sampled at 1000 frames/s.

Figure 3 and Figure 4 demonstrate sequential sEMG-GAF images from the NinaPro DB1 and CagpMyo DB-a datasets, respectively. Slight changes between adjacent frames of the sEMG image represent changes in the sEMG signal over time.

### 3.2. Construction of ConvNet Architecture

DCNN can effectively extract crucial spatial features from the data and has shown exemplary performance in several computer vision and pattern recognition competitions [35]. It is also a competitive classifier in many applications. A CNN-based architecture is essential to achieve good classification accuracy. Krizhevsky et al. [36] proposed AlexNet, which can enhance the learning capacity of a CNN by making the network deeper through various parameter learning strategies. The AlexNet architecture includes five convolution layers and three fully connected layers, which make a CNN applicable to classification tasks with diverse categories of images [37].

**Figure 5:**
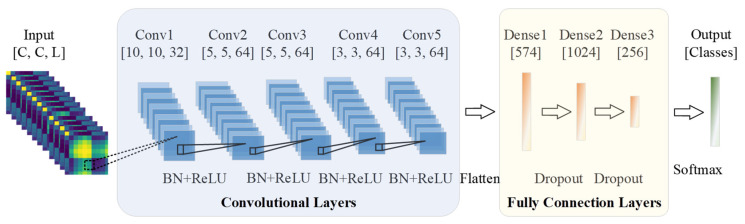
The architecture of the proposed five-layer convolutional neural network consists of an input layer, five convolutional layers, and three fully connected layers. The network’s input is a clip of a sequence of sEMG images with size *C* × *C* × *L*, where *C* is the number of sEMG sensor channels, and L is the duration of the sliding time window. Outputs of network are class labels of hand gestures. The dotted line between the input layer and the first convolutional layer denotes the kernel size. Therefore, the stride applied to the input layer should differ according to the size of an input image.

To design a classification network for the sEMG-GAF image, an AlexNet architecture is introduced to the proposed framework to construct an appropriate DL model for extracting crucial features from sEMG-GAF images. As shown in Figure 5, the proposed network consists of an input layer, five convolutional layers, and three fully connected layers. The size of the input layer should be adjusted according to the size of the input data. In our work, the input data size is *C* × *C* × *L*, where *C* is the number of sEMG sensor channels and *L* is the length of the sliding time window. The kernel size and stride applied on the input layer are resized to adapt to the following convolutional layers. For example, the size of the input is 10 × 10 × 20 under a sliding time window with a length of 200 ms from a dataset with 10 channels and a sampling rate of 100 Hz. Therefore, the convolutional kernel size will be set to 3 × 3 with a zero-padding size of 1 × 1, and the stride should be set to 1. In addition, a dropout strategy with a probability of 0.5 on the two fully connected layers is adopted to prevent overfitting. With AlexNet, we employ batch normalization instead of using max-pooling layers after the convolution operation on each convolutional layer to optimize the network. Batch normalization can enhance the model’s generalization ability to reduce the dependence on parameter initialization, accelerate network training, and reduce the computational cost.

### 3.3. Rationale of the GAF-CNN Method

We discuss the rationale of the GAF-CNN method. It is well known that CNNs can not only preserve the neighborhood relations and spatial locality of an input image in latent higher-level feature representations but also can scale well to realistic-sized high-dimensional images in terms of computational complexity, since the number of free parameters that describe their shared weights does not depend on the input dimensionality [20]. Although some works [18,19,31] have shown the effectiveness of CNN models on myoelectric pattern recognition, those methods directly rearrange the sEMG signals in an image form. However, they are not able to fully leverage the advantages of CNNs. Consequently, how to construct an excellent 2D representation of multichannel sEMG signals for CNN learning is the essential point.

**Figure 6:**
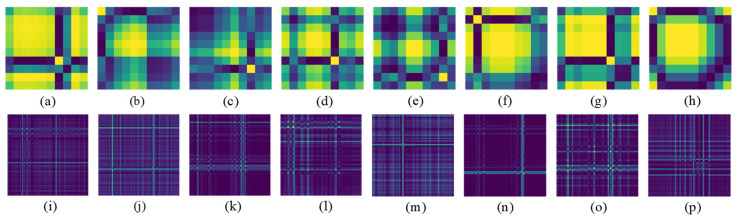
Demonstration of sEMG-GAF images of different gestures from NinaPro DB1 dataset (**a**–**h**) and CapgMyo DB-a dataset (**i**–**p**) on the specified moment. The gestures include thumb up as shown in (**a**) and (**i**), extension of index and middle, flexion of the others as shown in (**b**) and (**j**), flexion of ring and little finger, extension of the others as shown in (**c**) and (**k**), thumb opposing base of little finger as shown in (**d**) and (**l**), abduction of all fingers as shown in (**e**) and (**m**), fingers flexed together in a fist as shown in (**f**) and (**n**), pointing index as shown in (**g**) and (**o**), and adduction of extended fingers as shown in (**h**) and (**p**). The different colors in sEMG-GAF images indicate relationships between the instant values in different sensors. The color difference between the images of different gestures indicates the degree of differentiation of gestures.

We propose a GAF-CNN method to formulate sEMG-based gesture recognition as a multichannel sEMG image CNN-based classification problem. The GAF method is introduced for the 2D representation of multichannel sEMG signals. Unlike conventional GAF-based applications that encode time-series signals as images, we encode the multichannel sEMG signals on the channel dimension for two reasons. On one hand, the raw sEMG signals in each channel are nonstationary, nonlinear, stochastic, and unpredictable, making the correlations of time stamps during the process of raw sEMG signals not unsuitable for CNN-based gesture recognition. Our preliminary experimental results show that the performance of the CNN model is extremely poor and even cannot converge in most cases while applying the GAF method to convert sEMG signals on time series. On the other hand, the activation levels of different muscles will maintain relatively stable values while keeping the same hand gesture. Patterns of multichannel sEMG signals between instant values in different sensor channels should be easier to extract compared to the case in time series. Encoding multichannel signals as images can make the feature space more expressive by representing relationships between the values in different sensor channels rather than treating each channel independently. Figure 6 shows sEMG-GAF images obtained from different gestures at a specified moment. Different image texture patterns in sEMG images can be extracted easily. Therefore, encoding the instantaneous values as the image could be a good 2D representation of multichannel sEMG signals for CNN-based classification. 

In addition, we consider sEMG-GAF image construction from a mathematical rationale. As described in Section 3.1, we use the Gram matrix to construct the sEMG-GAF image. This is a helpful tool in linear algebra and geometry, which has been frequently used to compute the linear dependence of a set of vectors [20]. The mathematics of this method is intrinsically linked to the inner product and the corresponding Gram matrix. The inner product is an operation between two vectors to measure their similarity. In this work, the Gram matrix is an sEMG-GAF image. Since the channel index increases as the position moves from top-left to bottom-right, the correlation patterns between global channel information are encoded in the geometry of the matrix. As a result, patterns between gestures and the related muscles in sEMG-GAF images can be well extracted and represented by CNN models. It is noteworthy that the geometric characters of the sEMG-GAF image meet the classification capability of a CNN, which enables it to easily extract the patterns in the image.

To summarize, GAF-CNN employs the GAF method to exploit a 2D representation of multichannel sEMG signals for CNN-based classification. Compared with existing work [18,19,31] that directly rearranges the values of sEMG signals for classification, GAF representation has several advantages. First, it provides a 2D representation and makes the multichannel sEMG signals suitable as input for the CNN model. Second, it encodes the global channel correlations of sensors into local relationships and preserves the temporal dependency of the input image, so as to facilitate extraction by the CNN of gesture patterns in sEMG-GAF images. Finally, it is worth noting that instant values in the sensor channels are highly correlated with the collaboration of muscles, which are used for gesture control.

## 4. Experiments

### 4.1. Dataset Description

We discuss the four publicly available benchmark datasets used in the experiment: NinaPro DB1 [2], NinaPro DB2 [2], CapgMyo DB-a [38], and CSL-HDEMG [39]. For all datasets, the sEMG sensors were placed on various muscle locations on the upper limbs. We describe each dataset as follows:

1. NinaPro DB1 [2] consists of sEMG signals extracted from 27 intact subjects, captured with 10 sEMG electrodes (8 placed around the forearm, and 1 each on the main activity spots of the large flexor and extensor muscles of the forearm) at a 100 Hz sampling rate. The dataset has 52 gestures, each executed in 10 trials for 3–4 s.
2. NinaPro DB2 [2] consists of sEMG signals extracted from 40 intact subjects while performing finger gestures and grasping objects. The signals were sampled at a rate of 2000 Hz. During acquisition, subjects were asked to repeat movements with the right hand, each repetition lasting 5 s, followed by 3 s of rest. The protocol included six repetitions of 49 different movements (plus rest).
3. CapgMyo Db-a [38] is a frequently used high-density sEMG dataset, which was recorded with an 8 × 16 electrode grid wrapped around the right forearm, including eight isometric and isotonic finger gestures acquired from 18 healthy subjects. The 128-channel signals were band-pass filtered at 20–380 Hz with a sampling rate of 1000 Hz. Each subject performed 10 trials of each gesture, holding each for 3–5 s.
4. CSL-HDEMG [39] consists of high-density sEMG signals recorded at a sampling rate of 2048 Hz using an electrode array arranged in an 8 × 24 grid. The dataset includes 27 finger gestures performed by five subjects in five recording sessions, recording each gesture 10 times in each session.

### 4.2. Data sEMG-GAF Transformation

As discussed in Section 3.1, instantaneous sEMG signals with *C* sensor channels on each sampling moment were converted to an sEMG-GAF image with a size of *C* × *C* before training and prediction. Table 1 shows the sizes of the converted sEMG-GAF image for each dataset. Taking one instance, sEMG signals in NinaPro DB1 were captured with 10-channel sEMG electrodes. Therefore, each converted sEMG-GAF image had a size of 10 × 10, and the input size for the proposed model was 10 × 10 × 1. For sequential sEMG-GAF images, the interval of each frame was equal to the time segmentation of the sEMG signal series.

### 4.3. Experimental Setup

For each subject, we evaluated inter-session classification accuracies, where the proposed model was trained on 70% of the samples and evaluated on the remaining 30%. For the training samples, we used a data augmentation strategy that replaces 10% of each sample with data from other samples with a probability of 50%. In this way, random disturbance could be added to the training data to prevent overfitting. Moreover, since the network training requires a large-scale labeled dataset, we executed the data augmentation strategy three times.

To evaluate the performance of the GAF-CNN method, we followed the accuracy calculation rule applied in [29] and [30], i.e., using the instantaneous sEMG images for training and classification first, and then employing a majority voting strategy to calculate classification accuracies over the specified length time window, e.g., 150 ms and 300 ms. Therefore, the input sizes of NinaPro DB1, NinaPro DB2, CapgMyo Db-a, and CSL-HDEMG were 10 × 10 × 1, 12 × 12 × 1, 128 × 128 × 1, and 192 × 192 × 1, respectively. 

Since the GAF-CNN method can handle the input size of sEMG signals with different sensor channel amounts and various sliding windows as presented in Section 3.2, we also conducted experiments both using a sliding window strategy and a voting strategy for classification accuracy calculation on datasets captured with low sampling rates, i.e., NinaPro DB1. For NinaPro DB1, we used an input size of 10 × 10 × *L*, where the amount of the sensor channel is 10 and the *L* is the length of the sliding window of 10 ms, 100 ms, and 200 ms, corresponding to a single frame, 10 frames, and 20 frames of sEMG-GAF images, respectively. 

Based on the experimental results, the classification accuracy was calculated for each dataset as

(6) $$\mathrm{Classification}\;\mathrm{Accuracy}=\frac{\mathrm{Numbers}\;\mathrm{of}\;\mathrm{correct}\;\mathrm{classifications}}{\mathrm{Total}\;\mathrm{number}\;\mathrm{of}\;\mathrm{classifications}}\times 100.$$

The proposed GAF-CNN method was implemented based on the Keras 1.18.5 platform, written in Python and capable of running on top of TensorFlow. Experiments were conducted on a computer equipped with one Nvidia GPU GTX 3090 with 64 GB RAM, and an Intel CPU i9 9900 k. 

## 5. Results and Discussion

### 5.1. Role of Input Sizes

The proposed GAF-CNN method was analyzed with respect to the performance using sliding window and voting strategies. 

**Figure 7:**
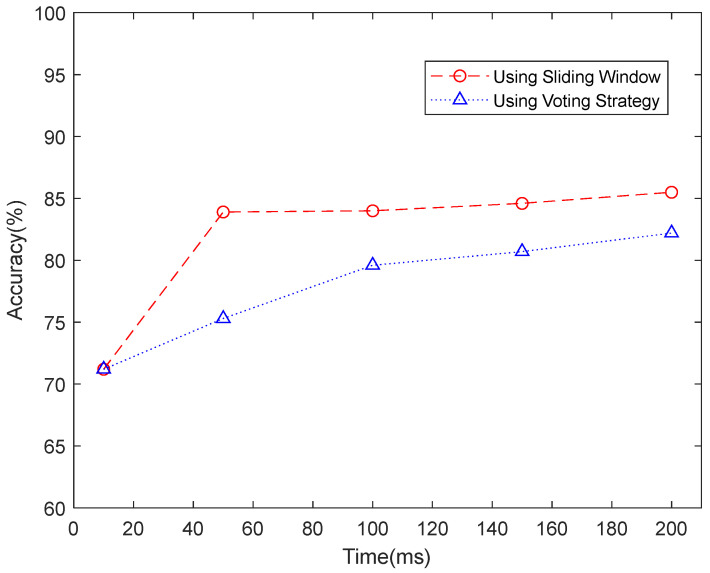
Recognition accuracy comparison between using sliding window and voting strategy in NinaPro DB1.

**Figure 8:**
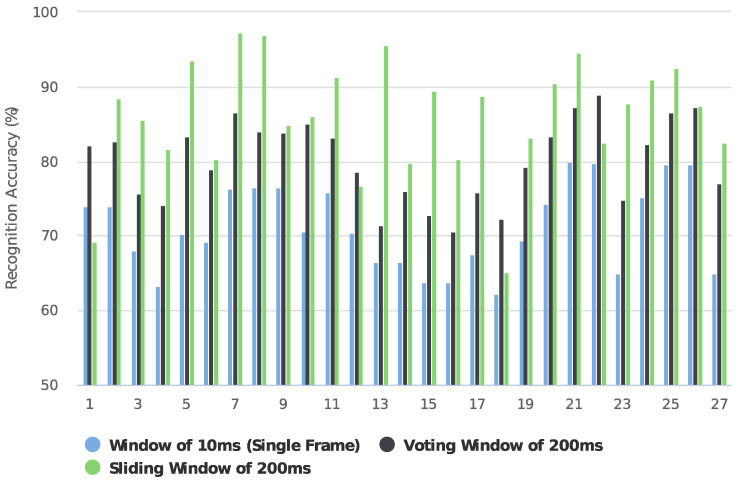
Recognition performances of 27 subjects when using single frame of 10 ms, voting time window of 200 ms, and sliding time window of 200 ms in NinaPro DB1.

For the low-density dataset, we tried both sliding windows and voting strategies. Figure 7 shows the recognition accuracy comparison between using sliding windows and voting strategies in NinaPro DB1. When using sliding windows of 10 ms, 50 ms, 100 ms, 150 ms, and 200 ms, the recognition accuracies of 71.2%, 83.9%, 84.0%, 84.6%, and 85.5% were achieved. Since the sampling frequency is 100 Hz, the recognition accuracy of 71.2% on a sliding window of 10 ms can be considered as instantaneous recognition accuracy. Based on the instantaneous recognition accuracy, we employed a majority voting strategy to re-calculate the accuracies over time windows of 50 ms, 100 ms, 150 ms, and 200 ms, by which we achieved accuracies of 75.3%, 79.6%, 80.7%, and 82.2%, respectively. Figure 8 further compares the accuracy of each subject when using a single frame, a majority voting time window of 200 ms, and a sliding segmented time window of 200 ms in NinaPro DB1. The average classification accuracy of 71.2% was achieved when using every single frame as one sample. Based on this result, we applied a majority voting strategy over 200 ms to calculate the accuracy for each subject, and an average accuracy of 80.1% was obtained. As a comparison, the classification accuracy achieved by the sliding segmentation time window of 200 ms was 85.5%, which is significantly higher than the case calculated by a voting time window of 200 ms using a single frame. As a result, we believe that the majority voting strategy based on a single frame could lose information on the time domain of the myoelectric pattern. Therefore, we only report the recognition accuracies based on the sliding window strategy for NinaPro DB1.

For the high-density datasets, we employed a voting strategy for recognition accuracy calculation as in the existing works [19,31], since there are too many frames for the input of a CNN model even in a small sliding window. For instance, the window length of 100 ms contains 100 frames of sEMG-GAF images in the CapgMyo DB-a dataset, which could be an unbearable burden for the input of the CNN model.

### 5.2. Training Process

**Figure 9:**
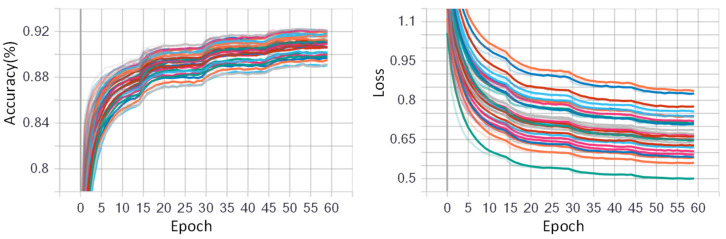
Training progress of fine-tuned GAF-CNN model for low-density dataset NinaPro DB1. The epoch number was set to 60 and different colors denote the 27 different subjects. The different color lines indicate different subjects.

**Figure 10:**
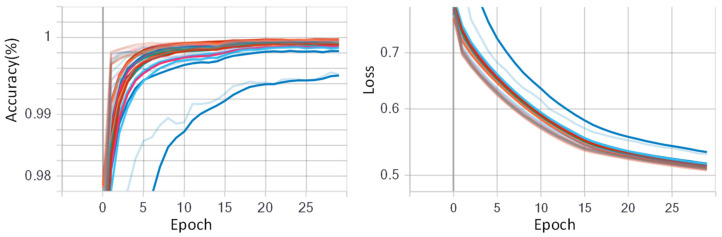
Training progress of fine-tuned GAF-CNN model for high-density dataset CapgMyo DB-a. The epoch number was set to 30 and different colors denote the 18 different subjects. The different color lines indicate different subjects.

The CNN model was initialized with randomized weights and trained using stochastic gradient descent (SGD) with a data batch size of 512 for all experiments. For fine-tuning the CNN model, the initial learning rate was assigned as 0.05 with a decay rate of 0.5. The initial learning rate was chosen large enough to accelerate the learning process in the beginning. The epoch number was set to 60 for dataset NinaPro DB1, and 30 for other datasets. Figure 9 and Figure 10 show the training progress of the fine-tuned CNN model for the low-density dataset NinaPro DB1 and the high-density dataset CapgMyo DB-a, respectively. The left shows the deviation i the accuracies on different training epochs, and the right shows the loss deviations in different training epochs. It is worth mentioning that 70% data were used for fine-tuning the AlexNet model, and the remaining 30% was used for testing the fine-tuned CNN model.

### 5.3. Evaluation on Recognition Accuracy

We evaluated the proposed GAF-CNN method on four publicly available datasets. The classification accuracies on a single frame of an instantaneous sEMG image were 71.0% on NinaPro DB1, 77.8% on NinaPro DB2, 45.0% on CSL-HDEMG, and 90.2% on CapgMyo DB-a, respectively. We found that data augmentation could significantly improve classification accuracies, achieving 71.2% on NinaPro DB1, 79.8% on NinaPro DB2, 46.3% on CSL-HDEMG, and 93.3% on CapgMyo Db-a, respectively, as well as an average improvement of 1.7%.

**Figure 11:**
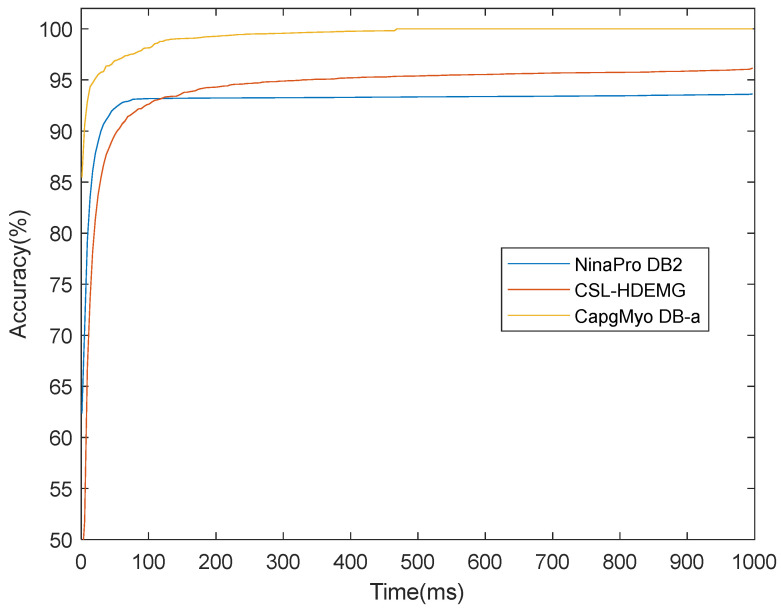
The recognition accuracies over different voting windows on high-density datasets NinaPro DB2, CSL-HDEMG, and CapgMyo DB-a. The recognition accuracies grow up when larger voting windows are applied.

Based on the instantaneous classification results, the recognition accuracies could be improved with a majority voting strategy. Figure 11 shows the recognition accuracies over different voting windows on high-density datasets of NinaPro DB2, CSL-HDEMG, and CapgMyo DB-a. It is clear that the recognition accuracies grew when larger voting windows were applied. For example, a classification accuracy of 93.3% was achieved on an instantaneous sEMG image on CapgMyo Db-a, and 99.5%, 99.7%, and 99.8% by majority voting over 40 ms, 150 ms, and 300 ms, respectively. 

### 5.4. Performance Comparison

To evaluate the performance of the proposed GAF-CNN method, we compared the experimental results with the state of the art as reported by previous work incorporating CNN models, which include a benchmark classifier with a handcrafted feature set, single-stream CNN by Atzori et al. [2], multi-stream CNN by Wei et al. [31], and 3D CNN by Chen et al. [32]. Single-stream CNN by Atzori et al. [2] and multi-stream CNN by Wei et al. [31] are popular 2D feature-based CNN methods, which employ the sEMG data as the direct input for the 2D CNN model. Table 2 presents the classification accuracies obtained by training and subsequently using the majority voting strategy to calculate the accuracies of the specified lengths of time windows on different sources of sEMG data.

Evaluations were also performed on the sparse sEMG datasets. The proposed GAF-CNN achieved classification accuracies of 83.9% over the window size of 100 ms and 85.5% over the window size of 200 ms, respectively, on NinaPro DB1. For NinaPro DB2, GAF-CNN achieved the classification accuracies of 79.0% and 80.2%, respectively. The experimental results show that the GAF-CNN method outperformed the CNN-based methods, including single-stream CNN [2] and multi-stream CNN by Wei et al. [31].

Regarding the evaluations performed on the high-density sEMG datasets, GAF-CNN showed comparable performances to all compared methods. Classification accuracies of 95.0% and 96.1% were achieved on CSL-HDEMG over window sizes of 150 ms and 300 ms, respectively. For CapgMyo DB-a, corresponding classification accuracies of 99.7% and 99.8% were achieved.

We also compared the proposed method with a 3D CNN-based method [32] on CSL-HDEMG and CapgMyo DB-a. The 3D CNN-based method [28] exploited the raw sEMG data with a cube size of l × 8 × 16 as the model input, where l was the sliding window length. Therefore, it could not identify the instantaneous sEMG signals, and it used significantly more computation when the sliding window became longer, while GAF-CNN achieved higher classification accuracy on the reported voting windows. For example, while using a sliding window length of 150 ms, GAF-CNN achieved a 4.3% (95.0% vs. 90.7%) higher accuracy on CSL-HDEMG, 1.1% (98.6% vs. 99.7%) higher on CSL-HDEMG, and 1.1% (98.6% vs. 99.7%) higher on CapgMyo DB-a, respectively, than those reported by Chen et al. [32].

## 6. Conclusions

We present a two-stage CNN-based architecture to effectively extract patterns from complex sEMG signals to improve the accuracy of myoelectric pattern recognition. Multichannel sEMG signals are encoded to sEMG-GAF image series by the GAF method so as to map the relationship between hand movements and active forearm muscle regions. Thus, the correlation patterns between individual muscles of specified hand movements could be thoroughly learned by the defined CNN model. To evaluate the effectiveness of the proposed method, we conducted extensive experiments on sparse and high-density sEMG datasets under sliding time windows and majority voting time windows, which showed that GAF-CNN achieves great performance on classification accuracy. Evaluation results on four publicly available sEMG datasets show that GAF-CNN outperforms the state-of-the-art CNN-based myoelectric pattern recognition methods.

In the future, we will improve the GAF-CNN-based myoelectric pattern recognition method in two ways: (1) A concise and practical deep CNN model for training and classification can be employed. We believe the classification accuracy can be improved by advanced DL techniques, such as deep transfer learning. (2) A CNN-based model can be employed to focus on the processing of sEMG data, which are temporal signals. Novel temporal models, such as R-CNN and LSTM [41], can be employed to exploit more time-domain features in myoelectric pattern recognition.

## Figures and Tables

**Table 1:** Size specification of the converted sEMG-GAF image for each dataset.

Datasets	Sensor Channels	sEMG-GAF Image Sizes	Frame Interval
NinaPro DB1	10	10 × 10	10 ms
NinaPro DB2	12	12 × 12	0.5 ms
CapgMyo Db-a	128	128 × 128	1 ms
CSL-HDEMG	192	192 × 192	0.49 ms

**Table 2:** Classification accuracies achieved by GAF-CNN when evaluated on publicly available sEMG datasets, along with the state of the art as reported by previous work incorporating CNN models. The first row indicates the in-use datasets, and the second row indicates the experimental conditions for sliding window lengths. The bold font indicates the best result under the same conditions in comparisons.

	NinaPro DB1		NinaPro DB2		CSL-HDEMG		CapgMyo DB-a	
	100 ms	200 ms	100 ms	200 ms	150 ms	300 ms	150 ms	300 ms
Benchmark classifier with handcrafted feature set	-	75.3% from [2]	-	75.2% from [2]	-	-	99.0% from [40]	-
Single-stream CNN by Atzori et al. [2]	-	66.6%	-	60.3%	-	-	99.5%	-
Multi-stream CNN by Wei et al. [31]	83.4%	85.0%	-	-	93.6%	95.4%	**99.7%**	**99.8%**
3D CNN by Chen et al. [32]	-	-	-	-	90.7%	-	98.6%	-
Proposed GAF-CNN	** 83.9% **	** 85.5% **	** 79.0% **	** 80.2% **	** 94.8% **	** 95.9% **	** 99.7% **	** 99.8% **

## Data Availability

Publicly available datasets were analyzed in this study. This data can be found here: http://ninaweb.hevs.ch/, http://zju-capg.org/research_en_electro_capgmyo.html, and https://www.uni-bremen.de/csl/forschung/gestenerkennung (all accessed on 10 December 2022).
